# Supplementary material for: Test cricketers score quickly during the ‘nervous nineties’: Evidence from a regression discontinuity design
Source: PLoS One. 2023 Jun 28;18(6):e0287700. doi: 10.1371/journal.pone.0287700 (PMC10306206; doi:10.1371/journal.pone.0287700)
Supplement: S1 Table — Note: Model for runs estimated using a multi-level mixed effects linear regression model. Models for boundaries and dismissals estimated using a multi-level mixed-effects logistic regression model. (DOCX) [file pone.0287700.s001.docx]

|  | **Runs** | | **Boundaries** | | **Dismissals** | |
| --- | --- | --- | --- | --- | --- | --- |
|  | **Coef. (95% CI)** | **P** | **Coef. (95% CI)** | **P** | **Coef. (95% CI)** | **P** |
| **Fixed effects** |  |  |  |  |  |  |
| I(Score ≥ 100) | -.18 (-.22 to -.14) | 0.0000 | -.49 (-.62 to -.37) | 0.0000 | .017 (-.18 to .21) | 0.8664 |
| Score - 100 | .011 (.0073 to .015) | 0.0000 | .034 (.021 to .046) | 0.0000 | -.0021 (-.009 to .0047) | 0.5379 |
| I(Score ≥ 100) x (Score - 100) | -.0036 (-.0092 to .002) | 0.2098 | -.014 (-.034 to .0055) | 0.1575 | .0049 (-.0066 to .016) | 0.4002 |
| (Score - 100)^2^ | .00024 (.00013 to .00035) | 0.0000 | .00078 (.00041 to .0012) | 0.0000 | -- | -- |
| I(Score ≥ 100) x (Score - 100)^2^ | -.00039 (-.00057 to -.00021) | 0.0000 | -.0012 (-.0019 to -.00056) | 0.0002 | -- | -- |
| Intercept | .72 (.69 to .75) | 0.0000 | -2.3 (-2.4 to -2.2) | 0.0000 | -4.4 (-4.5 to -4.2) | 0.0000 |
| **Random effects** |  |  |  |  |  |  |
| Variance: player intercept | .0087 (.0061 to .012) |  | .054 (.038 to .076) |  | .082 (.045 to .15) |  |
| Variance: match intercept | .0039 (.00036 to .042) |  |  |  |  |  |
| Variance: innings intercept | .014 (.0073 to .028) |  |  |  |  |  |
