# Supplementary material for: Test cricketers score quickly during the ‘nervous nineties’: Evidence from a regression discontinuity design
Source: PLoS One. 2023 Jun 28;18(6):e0287700. doi: 10.1371/journal.pone.0287700 (PMC10306206; doi:10.1371/journal.pone.0287700)
Supplement: S2 Table — Note: Model for runs estimated using a multi-level mixed effects linear regression model. Models for boundaries and dismissals estimated using a multi-level mixed-effects logistic regression model. (DOCX) [file pone.0287700.s002.docx]

|  | **Runs** | | **Boundaries** | | **Dismissals** | |
| --- | --- | --- | --- | --- | --- | --- |
|  | **Coef. (95% CI)** | **P** | **Coef. (95% CI)** | **P** | **Coef. (95% CI)** | **P** |
| **Fixed effects** |  |  |  |  |  |  |
| I(Score ≥ 100) | -.19 (-.24 to -.15) | 0.0000 | -.55 (-.71 to -.39) | 0.0000 | .089 (-.15 to .33) | 0.4751 |
| Score - 100 | .0092 (.002 to .016) | 0.0121 | .039 (.015 to .063) | 0.0015 | -.0089 (-.022 to .0045) | 0.1918 |
| I(Score ≥ 100) x (Score - 100) | .0033 (-.0069 to .013) | 0.5276 | -.0086 (-.044 to .027) | 0.6399 | .013 (-.0075 to .034) | 0.2125 |
| (Score - 100)^2^ | .00012 (-.0002 to .00044) | 0.4521 | .00095 (-.00015 to .0021) | 0.0900 | -- | -- |
| I(Score ≥ 100) x (Score - 100)^2^ | -.00057 (-.001 to -.000084) | 0.0214 | -.002 (-.0037 to -.00027) | 0.0235 | -- | -- |
| Intercept | .72 (.68 to .75) | 0.0000 | -2.3 (-2.4 to -2.2) | 0.0000 | -4.4 (-4.6 to -4.3) | 0.0000 |
| **Random effects** |  |  |  |  |  |  |
| Variance: player intercept | .0072 (.0048 to .011) |  | .05 (.03 to .076) |  | .13 (.075 to .23) |  |
| Variance: match intercept | .0096 (.0026 to .035) |  |  |  |  |  |
| Variance: innings intercept | .0087 (.0021 to .037) |  |  |  |  |  |
