# Supplementary material for: Test cricketers score quickly during the ‘nervous nineties’: Evidence from a regression discontinuity design
Source: PLoS One. 2023 Jun 28;18(6):e0287700. doi: 10.1371/journal.pone.0287700 (PMC10306206; doi:10.1371/journal.pone.0287700)
Supplement: Attachment — Submitted filename: Comments.docx [file pone.0287700.s003.docx]

Test cricketers score quickly during the ‘nervous nineties’: Evidence from a regression discontinuity design

The aim of this manuscript was to determine the psychological impact on performance strategies of batting in the 90’s in test match cricket. The authors have a good understanding of the game of cricket and the statistical approach used to assess performance through this period is through and the results initially surprising. Given this, I think more attention could be played to the explanation of the results. The manuscript is well written, although I have some concerns with the justification used to differentiate this analysis from that of ODI cricket. I think a better argument can be made by referencing the task related constraints that differ between the games. Some of this has been done in the discussion section and the conclusion.

General Comments:

Is there a reason for choosing this time period? Maybe it is obvious in that this is all the data that was available in ball-by-ball format, if this is the case it might be good to tell the reader this.

Is there a reason for including male and female data together? Would it not be worth examining the female data separately (or excluded) given a century is a much rarer event in this game? Also given the disproportionate number of female games compared to male games any effect in the female game would be washed out and the results likely the same as if you didn’t include them.

It is mentioned that previous work has analysed soring in the nineties in ODI cricket, but test cricket might provide a better context to examine how batting is impacted by pressure due to its prestige. I question whether it is “better”, a century is extremely important to a batter regardless of the format. In fact, you could say it is easier to score a century in test cricket as the game dictates you don’t have to score as quickly. I would suggest restructuring this paragraph (line 141-146) to reference the task related constraints that dictate the game be played differently to ODI cricket.

Specific Comments:

Line 71-75: Mentions numerous sport specific areas of retrospective studies that have analysed high-pressure moments, it might be good to reference these in this sentence.

Line 316: “Sorting out” seems a little colloquial…rephrase.

Line 325-328: It is mentioned that less potent bowlers present the opportunities for batters reaching 100 runs to bat more conservatively in ODI cricket. This is quite speculative, and I think a good argument could also be made for the opposite occurring.

Line 332-333: In the below sentence, consider changing “are” to “it is possible that one-day batters…”

“Consequently, one-day batters **are** better placed than test cricketers to manage the nineties as they would like (e.g., safely progressing to 100)”
