# Supplementary material for: Test cricketers score quickly during the ‘nervous nineties’: Evidence from a regression discontinuity design
Source: PLoS One. 2023 Jun 28;18(6):e0287700. doi: 10.1371/journal.pone.0287700 (PMC10306206; doi:10.1371/journal.pone.0287700)
Supplement: Attachment — Submitted filename: Response to Reviewers.docx [file pone.0287700.s004.docx]

We thank the academic editor and both reviewers for taking the time to read and consider our work and offering their constructive suggestions. From these comments, we have made several adjustments to the paper that we believe will improve its broader acceptance and appeal. These changes include providing more detail about the ball-by-ball dataset used, reporting a validation exercise that we performed to verify the data used, enhancing the argument used to justify an examination of this topic in test cricket, and the removal of some of the more speculative sentences. Below we provide specific responses to each reviewer comment with some more detailed answers to the questions raised by reviewer 2 in relation to our chosen analyses.

Reviewer 1:

Is there a reason for choosing this time period? Maybe it is obvious in that this is all the data that was available in ball-by-ball format, if this is the case it might be good to tell the reader this.

*More information has been added about the ball-by-ball data available, including that we used everything that was accessible at the time.*

Is there a reason for including male and female data together? Would it not be worth examining the female data separately (or excluded) given a century is a much rarer event in this game? Also given the disproportionate number of female games compared to male games any effect in the female game would be washed out and the results likely the same as if you didn’t include them.

*We did out of curiosity try the analysis using men-only, and as suggested, this had no impact on the findings. Given this, we left the relatively small sample of women in the dataset because we couldn’t see a good reason for publishing a male-only analysis. Likewise, a female-only analysis wasn’t feasible with the numbers available.*

It is mentioned that previous work has analysed scoring in the nineties in ODI cricket, but test cricket might provide a better context to examine how batting is impacted by pressure due to its prestige. I question whether it is “better”, a century is extremely important to a batter regardless of the format. In fact, you could say it is easier to score a century in test cricket as the game dictates you don’t have to score as quickly. I would suggest restructuring this paragraph (line 141-146) to reference the task related constraints that dictate the game be played differently to ODI cricket.

*As suggested, we have rewritten this section to suggest that test cricket is a good context to explore the impact of a century on batting and performance – including that it is free of some constraints of 50 over cricket. There is now less insinuation that it is necessarily the best context.*

Line 71-75: Mentions numerous sport specific areas of retrospective studies that have analysed high-pressure moments, it might be good to reference these in this sentence.

*These references have been added.*

Line 316: “Sorting out” seems a little colloquial…rephrase.

*We have rephrased.*

Line 325-328: It is mentioned that less potent bowlers present the opportunities for batters reaching 100 runs to bat more conservatively in ODI cricket. This is quite speculative, and I think a good argument could also be made for the opposite occurring.

*We have removed the sentence about the ability to undertake low risk batting when less potent bowlers are operating.*

Line 332-333: In the below sentence, consider changing “are” to “it is possible that one-day batters…”

“Consequently, one-day batters **are** better placed than test cricketers to manage the nineties as they would like (e.g., safely progressing to 100)”

*We have adopted this suggested wording.*

Reviewer 2:
Data, please explain how authors ensured the data validity, just downloading the data from the internet?

*We have added information about a data validity exercise we undertook. While the data we used was the only ball-by-ball dataset we could find, we were able to completely verify the data at the innings level (i.e., by comparing individual innings run totals in the dataset we used with an alternative cricket database).*

Does the RD model account/ control for the year-by-year performance? As a temporal series autocorrelation, regression to the mean and outliers should be included to improve the robustness of the model. Please clarify these issues.

*The results presented in the manuscript do not control for time effects. However, to clarify this issue, we have re-run all our models controlling for time. Time trends were not significant for two of the outcomes (runs per over, p = 0.405 and probability of being dismissed, p = 0.620). Time was significant for the probability of getting a boundary (p = 0.017), and the direction of the relationship suggested that the odds of getting a boundary had declined very slightly per year (OR = 0.993). Nonetheless, when we looked at the values for the other coefficients, none had changed between models that adjusted for year and those that didn’t. Our interpretation is that time is not a confounder in this case. That makes sense. It is difficult to see what temporal changes may have changed the association at the discontinuity, which we note, is different to a more general question about how scoring rates and risk of dismissals have changed over time. Issues like regression to the mean are interesting but not directly relevant here as (a) we use a subset of all test data – the data of those who scored between 70 and 130 runs and (b) all models are fit as multilevel models with runs nested innings, innings nested within matches, and matches nested within players. Finally, we are of the school of thought that outliers should not be removed as this has the potential to introduce bias into the study, and the data are what they are.*

Why conduct an RD with repeated measures? Probably a multilevel model with random factors and clustered variables would fit either for these repeated measures from the data. Please justify why this model was used and not the MLM, random forest, or neural network...

*In fact, we did fit multilevel models with random factors/clustered variables, with RD assessed using (a) a term that captured whether or not the century had been reached (<100 = 0; >= 100 = 1) and (b) the interaction of the batter’s proximity to a century and the binary century variable. In effect, we ran a multilevel regression discontinuity model. Where useful, quadratic terms were also included. This method with justification is explained in the Analysis section.*

- L235, please write 1,123
- L238, please write 1,394

*These have been changed.*

- Results and discussion, my main concern about both sections is how the evolution of years (performance) is related to the model and presented to justify how it affects along the years where training methods, physical and technical training, as well as psychological training have evolved and can be affecting the identified trends.

*We have covered this point above.*

- Conclusions, L393-396, please avoid conclusions that were not obtained from your data. This suggestion can be included in the discussion but not in the conclusion.

*This speculative sentence has been deleted.*
